# Reply to Benedik et al. Comment on “Jakše et al. Vegan Diets for Children: A Narrative Review of Position Papers Published by Relevant Associations. *Nutrients* 2023, *15*, 4715”

**DOI:** 10.3390/nu16111774

**Published:** 2024-06-05

**Authors:** Boštjan Jakše, Zlatko Fras, Nataša Fidler Mis

**Affiliations:** 1 Independent Researcher, 1000 Ljubljana, Slovenia; bj7899@student.uni-lj.si; 2 Division of Medicine, Centre for Preventive Cardiology, University Medical Centre, 1000 Ljubljana, Slovenia; zlatko.fras@kclj.si; 3 Faculty of Medicine, University of Ljubljana, 1000 Ljubljana, Slovenia; 4 Ministry of Health, 1000 Ljubljana, Slovenia

Benedik et al. [1] expressed several disagreements about our recent paper [2]. We would like to clarify the dilemmas they exposed.

*Dilemma* *1:*

“First, the statements in this article do not represent the professional opinion of the Slovenian Paediatric Society, the Slovenian Association for School, University and Adolescent Medicine, the Slovenian Association for Clinical Nutrition, the Association of Nutritionists and Dietitians and the Slovenian Nutrition Society”.

*Answer* *1:*

The insights and perspectives provided in our narrative review [2] were never intended to be and should not be construed as any kind of official position or endorsement by the Slovenian Paediatric Society, the Slovenian Association for School, University and Adolescent Medicine, the Slovenian Association for Clinical Nutrition, the Association of Nutritionists and Dietitians, or the Slovenian Nutrition Society. 

Our publication is not a position statement, but “A Narrative Review of Position Papers Published by Relevant Associations”, as clearly stated in the title “Vegan Diets for Children: A Narrative Review of Position Papers Published by Relevant Associations” [2].

The manuscript title (“A narrative review”), type of study (“a narrative review”), aim (“to include scientific/professional associations and expert group position statements or opinions regarding the suitability of VN diets for children and adolescents available in PubMed/Medline or published on the institutions’ websites of these expert groups”), and main objective (“to evaluate”, “to explore”, and “ to investigate”), presented in our manuscript [2] were formulated very clearly, so it cannot be interpreted as a narrative review of any particular association or society. 

Consequently, employing this recognized methodological framework significantly diminishes the likelihood of misinterpretation, making the criticisms levelled by the authors [1] not only unjustified, but also redundant. From our perspective, assertions of an undue institutional bias are unfounded.

*Dilemma* *2:*

“The Professional Expert Panel of Paediatrics, the highest professional body in Slovenia under the Ministry of Health, does not endorse vegan diet in the paediatric population, as there is no convincing evidence for this type of diet in the most vulnerable population of our society—infants, toddlers, children, adolescents and young adults. In fact, most recent guidelines recommend dietary supplementation in children eating a vegan diet [2,3,4,5,6,7], and two recently published meta-analyzes raise concerns about the certainty of the evidence and call for more and better-designed studies given the lack of high-quality data [8,9]”.

*Answer* *2:*

The inclusion of “the most recent guidelines ([2,3,4,5,6,7])” in the comment [1] is beyond the scope of our narrative review [2]. In addition, the reference “5: Müller, P. Vegan Diet in Young Children. Nestle Nutr Inst Workshop Ser. 2020, 93, 103–110.” is not a guideline, but a review of one single author [3].

The commentary by the authors highlights a unique viewpoint, advocating for dietary supplementation in children who follow vegan diets and urging the scientific community to conduct more meticulously designed research on the effects of vegan diets on children. This perspective aligns with the critical insights we presented in our comprehensive narrative review. Additionally, we had already acknowledged the importance of their reference [4] as a crucial cautionary note regarding vegan diets for children (see Figure 1 [2]). 

Furthermore, our study incorporated more than 30 of the most relevant position statements from professional associations and expert groups. Rather than summarizing their viewpoints, we chose to quote them chronologically, as detailed in Table 1 [2].

*Dilemma* *3:*

“The National Institute of Public Health in Slovenia has also prepared an updated version of the guidelines for healthy eating in educational institutions in Slovenia, which does not support a vegan diet [10]. These guidelines have already been approved by the Professional Expert Panel of Paediatrics and will be implemented in the 2024/25 school year”.

*Answer* *3:*

The comments provided by the authors [1] diverge from the thematic scope of our narrative review [2]. Their approach seems to introduce unrelated topics and generate a semblance of confusion. This intertwining of themes detracts from the objective analysis and clear exposition that characterize rigorous academic discourse.

*Dilemma* *4:*

“Furthermore, some of the statements are not in line with the position paper of the European Society of Paediatrics Gastroenterology, Hepatology and Nutrition [2]”.

*Answer* *4:*

Our manuscript appropriately cites the work “Complementary Feeding: A Position Paper by the European Society for Paediatric Gastroenterology, Hepatology, and Nutrition (ESPGHAN) Committee on Nutrition”, co-authored by Fewtrell, M.; Bronsky, J.; Campoy, C.; Domellöf, M.; Embleton, N.; Fidler Mis, N.; Hojsak, I.; Hulst, J. M.; Indrio, F.; Lapillonne, A.; and Molgaard, C. (“J Pediatr Gastroenterol Nutr”, 2017) [5]. 

It is important to note that N.F.M., one of our manuscript’s co-authors, made significant contributions to that position paper [5] and has co-authored an additional 23 position papers and review articles with the ESPGHAN, which highlights her substantial contributions to paediatric nutrition research [6,7,8,9,10,11,12,13,14,15,16,17,18,19,20,21,22,23,24,25,26,27,28].

*Dilemma* *5:*

“However, the corresponding author of this article, Nataša Fidler Mis, claims to be an employee of the Paediatrics hospital at the University Medical Centre Ljubljana, Slovenia. She has been appointed by the Prime Minister to chair the newly established Slovenian Strategic Council for Nutrition described elsewhere [13] and has requested that her contract be suspended during this time. Therefore, she is not employed by the Paediatrics hospital, and her statements in this article do not reflect the position of this institution”.

*Answer* *5:*

We acknowledge and value your observation. N.F.M. is currently employed by the Ministry of Health in Ljubljana, Slovenia. In parallel, she is on a temporary 12-month leave of absence from the Division of Paediatrics at the University Medical Centre, Ljubljana, where she has been employed for 20 years. In addition, it is her intention to formally request a modification in the manuscript [2] to reflect her sole affiliation with the Ministry of Health in Ljubljana, Slovenia.

*Dilemma* *6:*

“Furthermore, she has not disclosed her conflict of interest. Her spouse Gregor Mis is the managing director of the advertising company VITA media, which focuses on advertising pharmaceutical and food products. VITA media’s slogan on its website [14] is “The most effective medium when it comes to health”. Again, this is a clear indication of a potential conflict of interest that was not disclosed in accordance with Nutrients’ disclosure rules”.

*Answer* *6:*

Regarding the manuscript [2], N.F.M. hereby formally declares that she has no content-related, organizational, financial, or any other personal interests with VITA media. This declaration comprehensively addresses all facets of the research presented, spanning content, structural organization, and financial aspects, to affirm unequivocally that there was no potential for influence on or compromise of the study’s impartiality. VITA media did not have any involvement in the study design, data collection, analysis, interpretation, or drafting and interpretation of the manuscript.

*Dilemma* *7:*

Secondly, the first author of the article did not disclose his conflict of interest with Herbalife Nutrition, which has already been disclosed elsewhere [11]: Boštjan Jakše and Barbara Jakše (i.e., Boštjan Jakše’s wife) created the commercial whole-food, plant-based lifestyle program. Part of the supplemental whole-food, plant-based diet uses products from Herbalife Nutrition, from which Boštjan Jakše and Barbara Jakše receive royalty compensation. This clearly indicates a potential conflict of interest, that was not disclosed according to Nutrients’ disclosure policy.

*Answer* *7:*

Disclosure of Conflicts: The first author, B.J., comprehensively disclosed all potential conflicts of interest [29,30,31,32,33,34,35] that could be pertinent to this study [2]. 

Personal Relationships: It is important to clarify that Barbara J. has not been married to Boštjan J. for almost two years; addressing any concerns regarding personal biases in connection with his former wife is irrelevant.

Program Origin and Purpose: Contrary to the assertions made, the commercial whole-food, plant-based lifestyle program initiated by B.J. is not exclusively dedicated to a specific eating pattern, but rather promotes a healthy and active lifestyle. The program is officially registered with the Copyright Agency of Slovenia. Individuals can autonomously choose their eating patterns without restrictions, stigma, imposition, or exclusion. It is recommended that the majority of the diet should come from whole-food sources. It is critical to note that, as explicitly mentioned in our manuscript, only approximately 15% of the program’s participants strictly adhere to a plant-based diet [34].

Dietary Recommendations by Herbalife: Herbalife does not specifically endorse a vegan diet; instead, it advocates for a balanced omnivore diet, diverging from the claims presented in the commentary [36,37,38].

Funding and Study Design: Herbalife has not provided funding for any of the studies led by the first author or any of the authors of the manuscript [2]. Herbalife was not involved in the study’s design, data collection, analysis, interpretation, or in the drafting and preparation of the manuscript [2].

Research Funding: Unlike some of the authors of the comment [1], B.J. has never received research funding from any commercial entity for his studies, further dispelling any notions of bias or undue influence.

Rejection of Unfounded Claims: B.J. rejects the unjustified and inappropriate allegations made by the comment’s authors [1]. These claims lack evidential support and falsely question the integrity of our research.

Conflicts of Interest of Commentary Authors: Finally, several of the authors of the comment [1] have failed to disclose their own conflicts of interest.

*Dilemma* *8:*

“Furthermore, the same author claims to be an independent researcher, which is not the case. During his PhD process, the members of the first appointed committee resigned because they considered his research unclear, and again he initially failed to disclose a conflict of interest [12]”.

*Answer* *8:*

B.J. categorically denies these allegations, emphasizing their complete lack of foundation. The doctoral thesis authored by the narrative review’s lead writer is recognized for its exceptional quality [39]. This thesis is supported by six scholarly articles (surpassing the requisite minimum of one article indexed in the PubMed database, with a total of two required) that were published in esteemed scientific journals and have garnered significant citations [29,30,31,32,33,34,35]. It is pertinent to mention that all manuscripts disclosing any conflict of interest were comprehensively included in the doctoral dissertation. Furthermore, details regarding the company were meticulously documented in the abstracts, available in Slovenian and English languages. B.J. contends that this level of disclosure ranks the thesis among the most transparent works submitted to this institution [39].

To further substantiate the falsity of the preceding statements, the first author, E.B. [1], who was actually the chair of the doctoral committee of B.J., gave a positive evaluation of the doctoral thesis. The corresponding author, T.B. [1], who withdrew from the committee during the PhD process, stated that he would not participate in a doctoral dissertation committee because it lacked a randomized, double-blind, placebo-controlled nutritional study. It is important to note that such a study does not exist, but it could be conducted using dietary supplements, extracts, and pharmacological studies. Additionally, the corresponding author, T.B. [1], disapproved of a dissertation committee incorporating more than one change, as seen in B.J.’s case involving nutrition, physical activity, and support systems, as part of the intervention. This information is documented in the Student Office for study programs on the third level of the Faculty of Biotechnology and in the legal department of the University of Ljubljana under two bookmarks.

For the reader’s understanding, it is important to note that the authors of the comment [1] have previously disclosed several conflicts of interest [40,41,42,43,44,45,46,47,48,49,50,51,52,53]; however, on several occasions, they failed to disclose any conflict of interest, indicating an inconsistency in their approach [54,55,56,57,58,59,60].

*Dilemma* *9:*

“Consequently, the literature included in the Narrative Review may lead to a biased view of the vegan diet, particularly in the paediatric population for whom there is no clear evidence to support this diet [5,6,7,8,9]”.

*Answer* *9:*

In our narrative review [2], as well as through the articulated positions of paediatricians in Slovenia concerning vegan diets for children, we underscored the imperative for more high-quality scientific research and the unbiased interpretation of results obtained (graphical abstract) [2].

*Dilemma* *10:*

“Finally, the authors of the manuscript are members of the National Strategic Council for Nutrition established by the Slovenian government, with most members of the council declaring themselves to be vocal supporters of a vegan diet. Furthermore, it is important to point out that the authors are not paediatricians and therefore their knowledge of the possible harmful consequences of an exclusively vegan diet for growing children may be limited. Therefore, this article should be read as a political manifesto rather than a scientific treatise”.

*Answer* *10:*

The authors [1] have made inaccurate allegations regarding the Strategic Council for Nutrition (SCN). The SCN is a consultative entity established by the Prime Minister, not by the Slovenian government. The SCN is characterized by its multidisciplinary composition, including representatives from (1) various ministries responsible for health, labor, family, social affairs, equal opportunities, education, science, sport, agriculture, forestry, food, environment, climate, energy, and digital transformation; (2) academic institutions such as the Biotechnical Faculty, Faculty of Medicine, Faculty of Social Sciences, and Faculty of Pharmacy; (3) other institutions and associations (e.g., the National Institute of Public Health of Slovenia, Umanotera—the Slovenian Foundation for Sustainable Development, and the Association of Nutritionists of Slovenia, mainly comprising food technologists and nutrition managers in educational institutions), as well as (4) private sector researchers focusing on nutrition and health, and advocates for local self-sufficiency [61,62]. It is noteworthy that the unified perspective of the SCN, as highlighted in our recent publication, advocates for an omnivorous diet, and particularly emphasizes health and sustainability, achieved through the reduction of food waste, and the promotion of healthier food accessibility for all [63]. In reference to the assertion of being “vocal supporters of a vegan diet”, we estimate that over 95% of SCN members adhere to an omnivorous diet.

Furthermore, the SCN’s alignment with global trends, such as reducing food waste, observing World Days dedicated to chronic noncommunicable diseases (NCDs), promoting nutrition and physical activity, fostering organic food production, ensuring national security in food supply, advocating for sustainable food consumption, implementing taxation on unhealthy food, alcohol, tobacco products, and sugar-sweetened beverages (SSBs), and adopting exemplary practices from abroad, is evident in publicly available publications meticulously referenced with scientific citations [61].

In addition, the scientific and professional activities of the SCN are also evident through its exemplary cooperation with the regional World Health Organization. This collaboration includes support for the taxation of unhealthy foods, sugar-sweetened beverages (SSBs), alcohol, and tobacco (in November 2023), as well as the development of new Slovenian food-based dietary guidelines (expert meeting in February 2024). Furthermore, the framework and conceptual design for a contemporary, cross-sectional (and potentially prospective) nationwide epidemiological study of population-level nutrition-related health indicators and disease burden (with a focus on chronic NCDs) were presented at, and supported by, the SCN.

In addition, the assertion that the authors’ lack of paediatric specialization (which is indeed true) limits their understanding of the potential harm of vegan diets to children diminishes the scientific integrity of the narrative review. Furthermore, such a declaration undermines the professional scientific merits of the individual authors of the review [2], particularly the second author, Z. F. Notably, Z. F. is a full professor and a long-standing specialist physician and consultant, with expertise in internal medicine, cardiology, and vascular medicine. He has held and currently holds important roles within the Slovenian medical community [64].

This narrative review’s objective scrutiny of vegan nutrition for children does not automatically imply a risk to child development, contrary to suggestions made. The call for paediatricians to engage in comparative nutritional research underscores the need for evidence-based analysis over speculative critique [2].

It is fundamental to emphasize that the authors of the narrative review [2], who are also members of the SCN, maintain no ties with political entities or interest groups, ensuring their neutrality. The SCN serves as a specialized advisory committee without political or professional executive mandates. Its advisories are presented to the Prime Minister as non-binding recommendations, reflecting its consultative role [61,62].

## Figures and Tables

**Figure 1:**
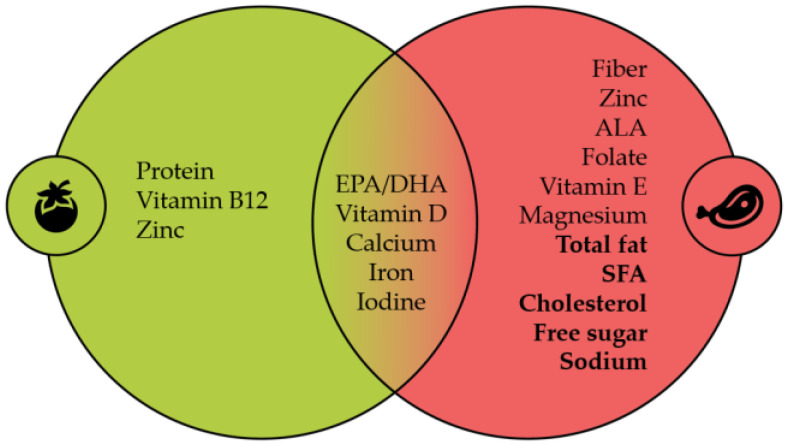
Green: VN diet, red: OM diet, nonbold: risk of inadequacy, bold: risk of excess intake. The data summarize the included studies and the latest systematic review.
